# Novel Arginine-containing Macrocyclic MMP Inhibitors: Synthesis, ^99m^Tc-labeling, and Evaluation

**DOI:** 10.1038/s41598-018-29941-2

**Published:** 2018-08-03

**Authors:** Yunpeng Ye, Jakub Toczek, Kiran Gona, Hye-Yeong Kim, Jinah Han, Mahmoud Razavian, Reza Golestani, Jiasheng Zhang, Terence L. Wu, Mousumi Ghosh, Jae-Joon Jung, Mehran M. Sadeghi

**Affiliations:** 1 Cardiovascular Molecular Imaging Laboratory, Section of Cardiovascular Medicine and Yale Cardiovascular Research Center, Yale University School of Medicine, New Haven, CT USA; 2 Veterans Affairs Connecticut Healthcare System, West Haven, CT USA; 3 Yale West Campus Analytical Core, Yale University, West Haven, CT USA

## Abstract

Matrix metalloproteinases (MMPs) are involved in tissue remodeling. Accordingly, MMP inhibitors and related radiolabeled analogs are important tools for MMP-targeted imaging and therapy in a number of diseases. Herein, we report design, synthesis, and evaluation of a new Arginine-containing macrocyclic hydroxamate analog, RYM, its hydrazinonicotinamide conjugate, RYM1 and ^99m^Tc-labeled analog ^99m^Tc-RYM1 for molecular imaging. RYM exhibited potent inhibition against a panel of recombinant human (rh) MMPs *in vitro*. RYM1 was efficiently labeled with ^99m^TcO_4_^−^ to give ^99m^Tc-RYM1 in a high radiochemical yield and high radiochemical purity. RYM1 and its decayed labeling product displayed similar inhibition potencies against rhMMP-12. Furthermore, ^99m^Tc-RYM1 exhibited specific binding with lung tissue from lung-specific interleukin-13 transgenic mice, in which MMP activity is increased in conjunction with tissue remodeling and inflammation. The results support further development of such new water-soluble Arginine-containing macrocyclic hydroxamate MMP inhibitors for targeted imaging and therapy.

## Introduction

Matrix metalloproteinases (MMPs) are a family of zinc-dependent endopeptidases, best known for their role in the degradation of extracellular matrix components. In part based on their enzymatic activity, these secreted or membrane-bound proteins may be classified into several classes i.e., collagenases, gelatinases, stromelysins, membrane-type MMPs, and others. As such, MMPs are involved in complex physiological and biological processes such as, tissue remodeling, wound healing, embryotic development, angiogenesis, cell adhesion, and proliferation. Tissue MMP activity is regulated by the MMP activation state and endogenous inhibitors such as tissue inhibitor of metalloproteinases (TIMPs). Many diseases, such as cancer, atherosclerosis, stroke, aortic aneurysm, cardiomyopathy, arthritis, and chronic obstructive pulmonary disease are associated with dysregulated expression and activation of MMPs^1–3^. Therefore, MMPs are attractive targets for developing new imaging and therapeutic strategies in various diseases.

Over the past decades, various MMP inhibitors including some synthetic small molecules have been developed^4–6^. MMP inhibitors labeled with radioisotopes and fluorophores have been investigated for imaging and characterization of MMP activity *in vivo*^7–15^. Accordingly, MMP-targeting has emerged as a promising approach for early detection, risk stratification, and therapy in a number of diseases. However, existing agents are suboptimal for *in vivo* use and novel MMP inhibitors and related imaging agents with improved properties and targeting activities are in high demand.

**Figure 1:**
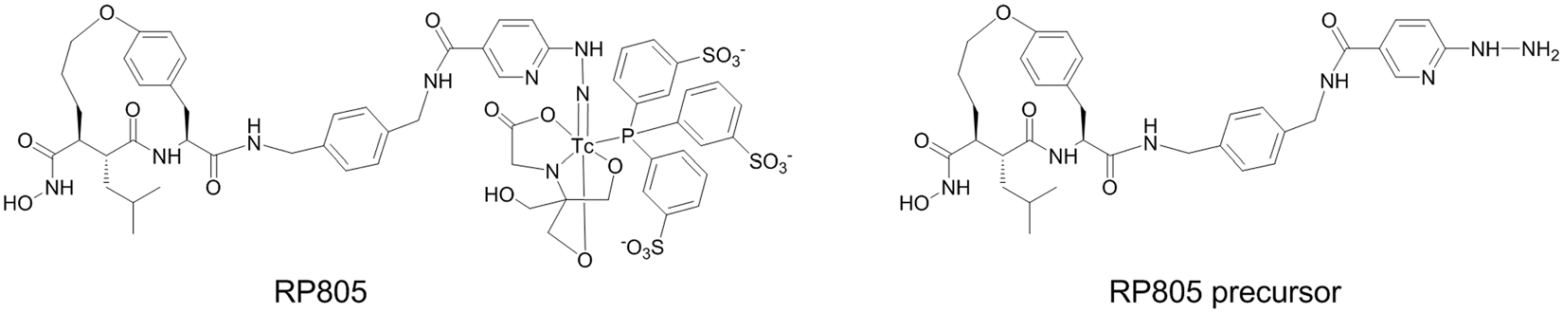
Structures of MMP-targeted SPECT imaging agent RP805 and its precursor.

RP805, is a ^99m^Tc-labeled macrocyclic hydroxamate MMP inhibitor (Fig. 1). Over the past decade, this tracer has been studied for MMP-targeted single-photon emission computed tomography (SPECT) imaging of cardiovascular and other pathologies in several preclinical models^16–22^. In vascular pathology, such as atherosclerosis and aneurysm, tissue uptake of RP805 correlates with vessel wall MMP activity and inflammation. Accordingly, this agent may be used to characterize atherosclerotic lesions, and evaluate aortic aneurysm progression and rupture risk. However, RP805 has a relatively prolonged blood circulation which may not be optimal for early time imaging of vascular diseases. In addition, the poor water solubility of RP805 precursor has impeded blocking studies *in vivo* at suitable concentrations. Therefore, we sought to pursue new macrocyclic hydroxamate analogs and related imaging agents with improved physicochemical properties for *in vivo* MMP-targeted imaging and therapy, with the ultimate goal of future clinical translation.

**Figure 2:**
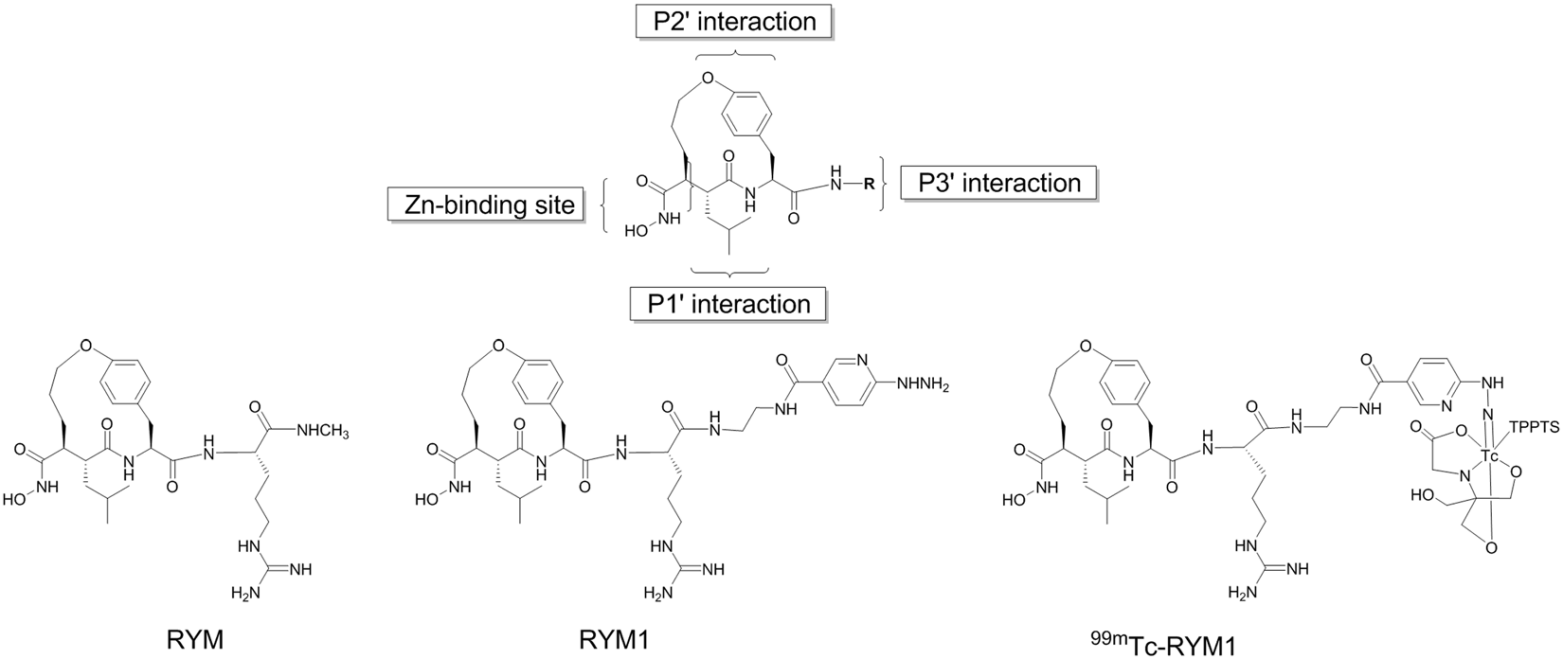
Molecular design of novel arginine-containing hydroxamate-based MMP inhibitors. RYM, RYM1, and SPECT imaging agent ^99m^Tc-RYM1.

Previous studies performed on anti-succinate-based hydroxamic acids have elucidated the structural requirements of this class of inhibitors for anti-MMP activity, which include a hydroxamate as a zinc-binding site, as well as α, P1′, P2′ and P3′ substituents^1^. The preferred conformation of this type of inhibitors displays α and P2′ substituents on the same side with close proximity spatially, which can be cyclized as shown in RP805 and its precursor. Wide variations of P3′ substituent can be tolerated. Based on this structure-activity relationship (Fig. 2), we designed a new macrocyclic hydroxamate-based analog, RYM, containing an Arginine (Arg) residue at P3′ position for improving the hydrophilicity. Furthermore, we designed an analog, RYM1, containing a hydrazinonicotinamide (HYNIC) as a precursor for ^99m^Tc-labeling to form ^99m^Tc-RYM1 for pilot studies. Herein, we report the related synthesis, ^99m^Tc labeling, MMP inhibition, and other evaluations *in vitro* and *ex vivo*. *In vivo* evaluation of ^99m^Tc-RYM1, including biodistribution, imaging and binding specificity in murine models of carotid and aortic aneurysm, is reported elsewhere^23^.

## Results

### Synthesis

**Figure 3:**
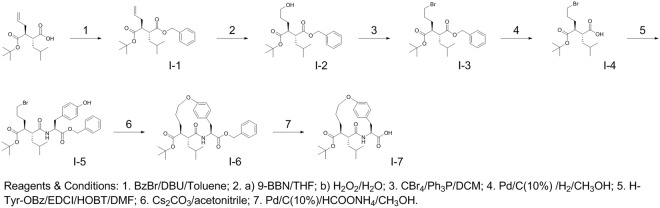
Synthesis of an anti-succinate-based macrocyclic acid intermediate (**I-7**).

As shown in Fig. 3, the synthetic strategy includes an initial synthesis of a protected anti-succinate-based macrocyclic acid analog (**I-7**)^24,25^. The synthesis was started from an anti-succinic acid derivative, i.e. (2R,3S)-3-(tert-butoxycarbonyl)-2-isobutylhex-5-enoic acid which was first converted into its benzyl ester (**I-1**) in the presence of 1,8-diazabicyclo[5.4.0]undec-7-ene (DBU) and benzyl bromide in toluene. Hydroboration of **I-1** by 9-borabicyclo[3.3.1]nonane (9-BBN) in tetrahydrofuran (THF), followed by oxidation with H_2_O_2_, gave the hydroxyl derivative (**I-2**). The conversion of **I-2** into its bromide derivative (**I-3**) was achieved using CBr_4_ and triphenylphosphine (Ph_3_P) in dichloromethane (DCM). The benzyl ester group of **I-3** was removed by catalytic hydrogenation to give its acid analog (**I-4**). Coupling of **I-4** with L-tyrosine benzyl ester in the presence of 1-ethyl-3-(3-dimethylaminopropyl) carbodiimide (EDCI) and hydroxybenzotriazole (HOBT) in N,N-dimethylformamide (DMF) gave the anti-succinate-tyrosine benzyl ester (**I-5**). Cyclization of **I-5** in the presence of Cs_2_CO_3_ in anhydrous acetonitrile gave the protected anti-succinate macrocyclic derivative (**I-6**). Finally, the macrocyclic acid analog (**I-7**) was obtained by removing the benzyl group of **I-6** in the presence of 10% palladium on carbon (Pd/C) and HCOONH_4_ in methanol.

**Figure 4:**
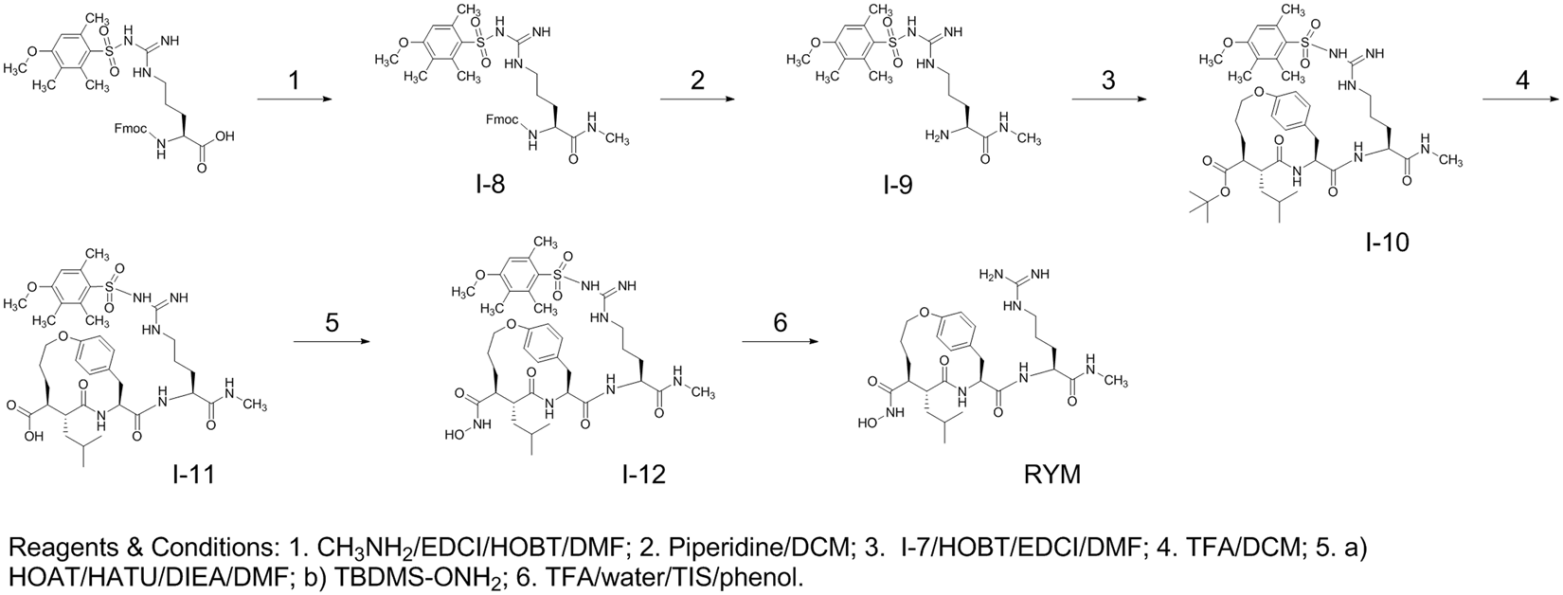
Synthesis of an Arg-containing anti-succinate-based macrocyclic hydroxamate, RYM.

As depicted in Fig. 4, we used Fmoc-Arg(Mtr)-OH as the starting material because the protecting group Mtr is stable in trifluoroacetic acid (TFA) solution compared with another commonly used protecting group Pbf in Fmoc-Arg(Pbf)-OH^26^. Therefore, Fmoc-Arg(Mtr)-OH was first reacted with CH_3_NH_2_ in the presence of EDCI/HOBT in DMF to give Fmoc-Arg(Mtr)-NHCH_3_ (**I-8**). De-protection of **I-8** with piperidine in DCM gave H-Arg(Mtr)-NHCH_3_ (**I-9**). The reaction between **I-7** and **I-9** in the presence of EDCI/HOBT afforded the Arg(Mtr)-containing macrocycle derivative (**I-10**), followed by de-protection of **I-10** with TFA to give the carboxylic acid analog (**I-11**). The pre-activation of **I-11** in the presence of HOAT, HATU, and DIEA in DMF, followed by reaction with O-(*tert*-butyldimethylsilyl)hydroxylamine (TBDMS-ONH_2_) gave **I-12**. Finally, the target compound RYM was obtained by de-protection of **I-12** in a solution of TFA, water, TIS, and phenol.

**Figure 5:**
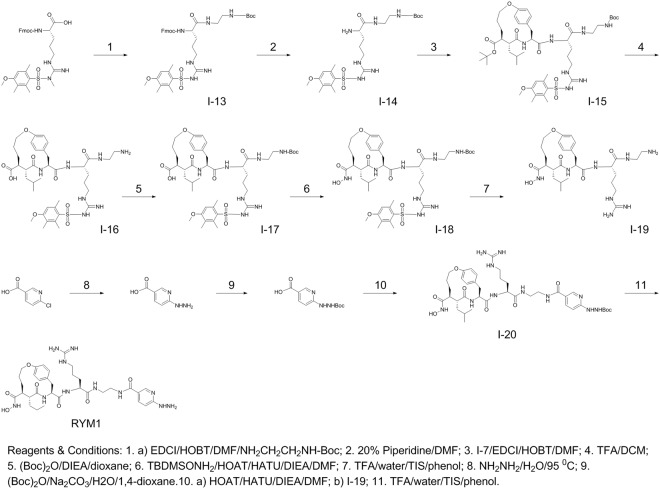
Synthesis of an Arg-containing macrocyclic hydroxamate HYNIC conjugate, RYM1.

We used a similar strategy for synthesis of the HYNIC conjugate (**2**). Therefore, Fmoc-Arg(Mtr)-OH was first reacted with NH_2_CH_2_CH_2_NH-Boc in the presence of EDCI/HOBT in DMF to give Fmoc-Arg(Mtr)- NH_2_CH_2_CH_2_NH-Boc (**I-13**) as depicted in Fig. 5. De-protection of **I-13** with 5% piperidine in DCM gave H-Arg(Mtr)- NH_2_CH_2_CH_2_NH-Boc (**I-14**). The reaction between **I-7** and **I-14** in the presence of EDCI/HOBT afforded the Arg(Mtr)-containing macrocycle derivative (**I-15**), followed by de-protection of **I-15** with TFA to give the carboxylic acid analog (**I-16**). **I-16** was reacted with (Boc)_2_O in aqueous solution of Na_2_CO_3_ to form the Boc-protected analog **I-17**. The pre-activation of **I-17** in the presence of HOAT, HATU, and DIEA in DMF, followed by reaction with TBDMS-ONH_2_ gave **I-18**. **I-18** was de-protected in a solution of TFA, water, TIS, and phenol to give **I-19**. Boc-HYNIC was prepared in two steps^27^ and activated in the presence of HATU, HOAT, DIEA, and DIEA in DMF. The activated Boc-HYNIC-OAT was reacted with **I-19** to give **I-20** which was de-protected in a solution of TFA, TIS, phenol, and water to give the desired HYNIC conjugate, RYM1.

The two crude products (RYM and RYM1) were purified by semi-preparative HPLC using aqueous acetonitrile as mobile phase to give the desired fractions as identified by LC-MS. The final products were obtained as white powder from lyophilization, which should be their corresponding TFA salts of the guanidine group. Both were further identified by analytical HPLC, high resolution mass spectroscopy (HR-MS, Supplemental Figure) and ^1^H/^13^C NMR analysis.

### MMP inhibition assays

MMP inhibition assays were carried out based on the kinetic effects of an inhibitor on the MMP-mediated catalytic cleavage of a fluorogenic substrate, namely Mca-Lys-Pro-Leu-Gly-Leu-Dpa-Ala-Arg-NH_2_, as reported by others previously^9,23,28^. In the first set of studies, the inhibitory effect of RYM was evaluated against 5 activated recombinant human (rh)MMPs, including rhMMP-2, rhMMP-7, rhMMP-9, rhMMP-12, and rhMMP-13. GM-6001, a broad-spectrum MMP inhibitor, was also tested for comparison. As expected, GM-6001 showed potent inhibitory activities against the five rhMMPs without significant selectivity and with the K_i_ values close to those reported in the literature (Table 1). RYM also had potent MMP inhibition with K_i_ values at nM concentrations. Unlike GM-6001, RYM showed modest selectivity against MMP-12 among the 5 tested rhMMPs.

**Table 1:** Inhibition constants (K_i_) of RYM and GM 6001.

K_i_* (nM)	rhMMP-2	rhMMP-7	rhMMP-9	rhMMP-12	rhMMP-13	TACE
RYM	5.8 ± 0.4	17.2 ± 0.1	20.4 ± 2.0	1.0 ± 0.5	15.3 ± 5.4	4.9 ± 1.3
GM-6001	0.5 ± 0.1	0.4 ± 0.0	1.2 ± 0.0	0.6 ± 0.0	0.3 ± 0.0	113.1 ± 4.1

*K_i_ values represent the mean ± SD of three experiments.

Tumor necrosis factor α converting enzyme (TACE) structurally belongs to the ADAM (short for a disintegrin and metalloproteinase) family of proteases, but its catalytic site is similar with that of MMPs^25^. Therefore, we evaluated RYM and GM-6001 for inhibition against TACE using rhTACE and the fluorogenic peptide substrate, Mca-Pro-Leu-Ala-Gln-Ala-Val-Dpa-Arg-Ser-Ser-Ser-Arg-NH_2_. RYM had strong TACE inhibition with a K_i_ value of 4.9 nM, which is close to the K_i_ value of rhMMP-2 inhibition. Interestingly, GM-6001 showed much weaker inhibition against rhTACE (K_i_ 113.1 nM) than rhMMPs. Next, we evaluated RYM1 for its inhibition against rhMMP-12 and TACE. This showed K_i_ values of 2.2 ± 0.5 nM (rhMMP-12)^23^ and 8.6 ± 2.2 (TACE). As such, both RYM and RYM1 had similar inhibitory potency against rhMMP-12 and TACE.

### Radiochemistry

**Figure 6:**
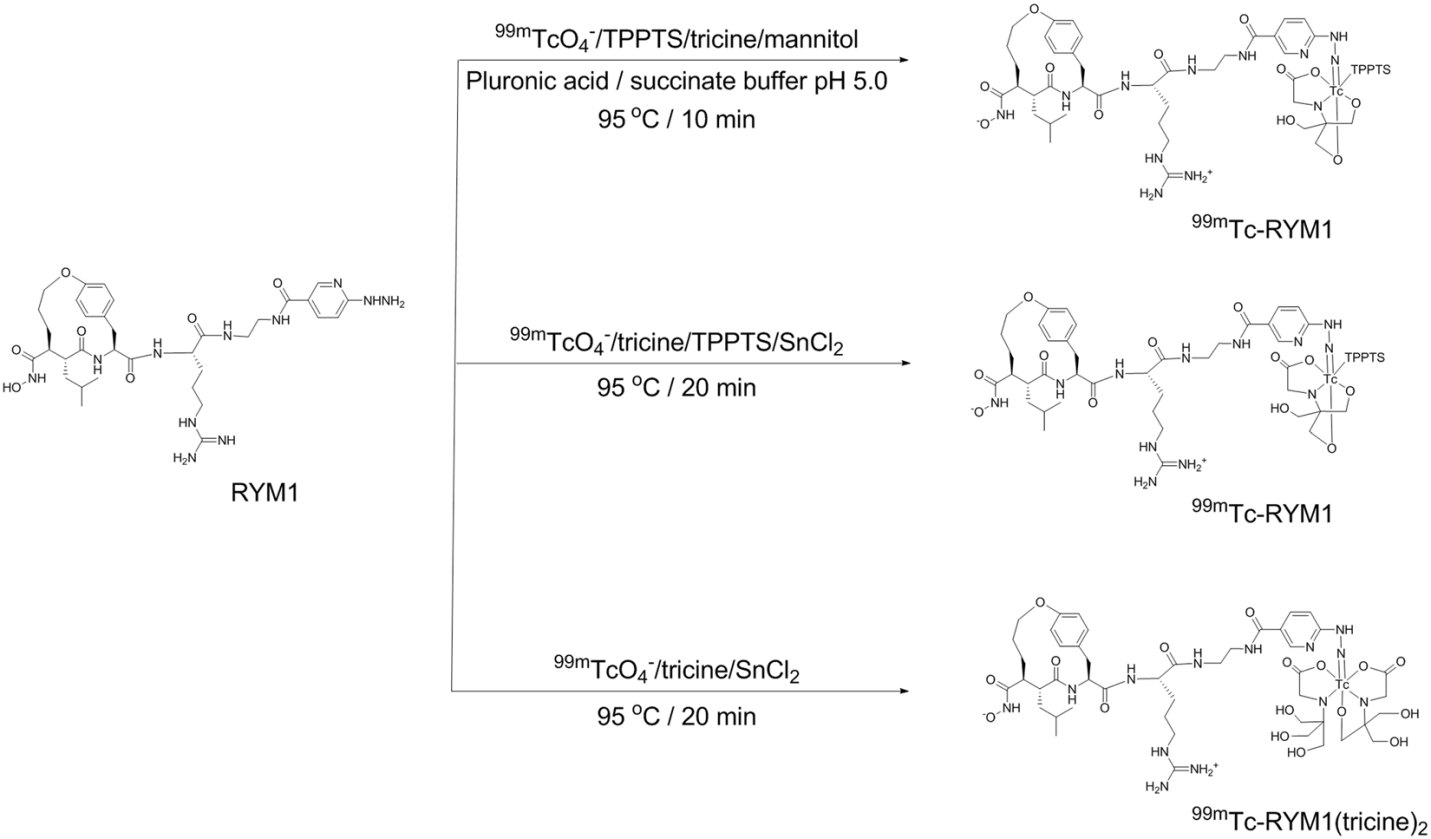
^99m^Tc-labeling of RYM1 using 3 different methods.

**Figure 7:**
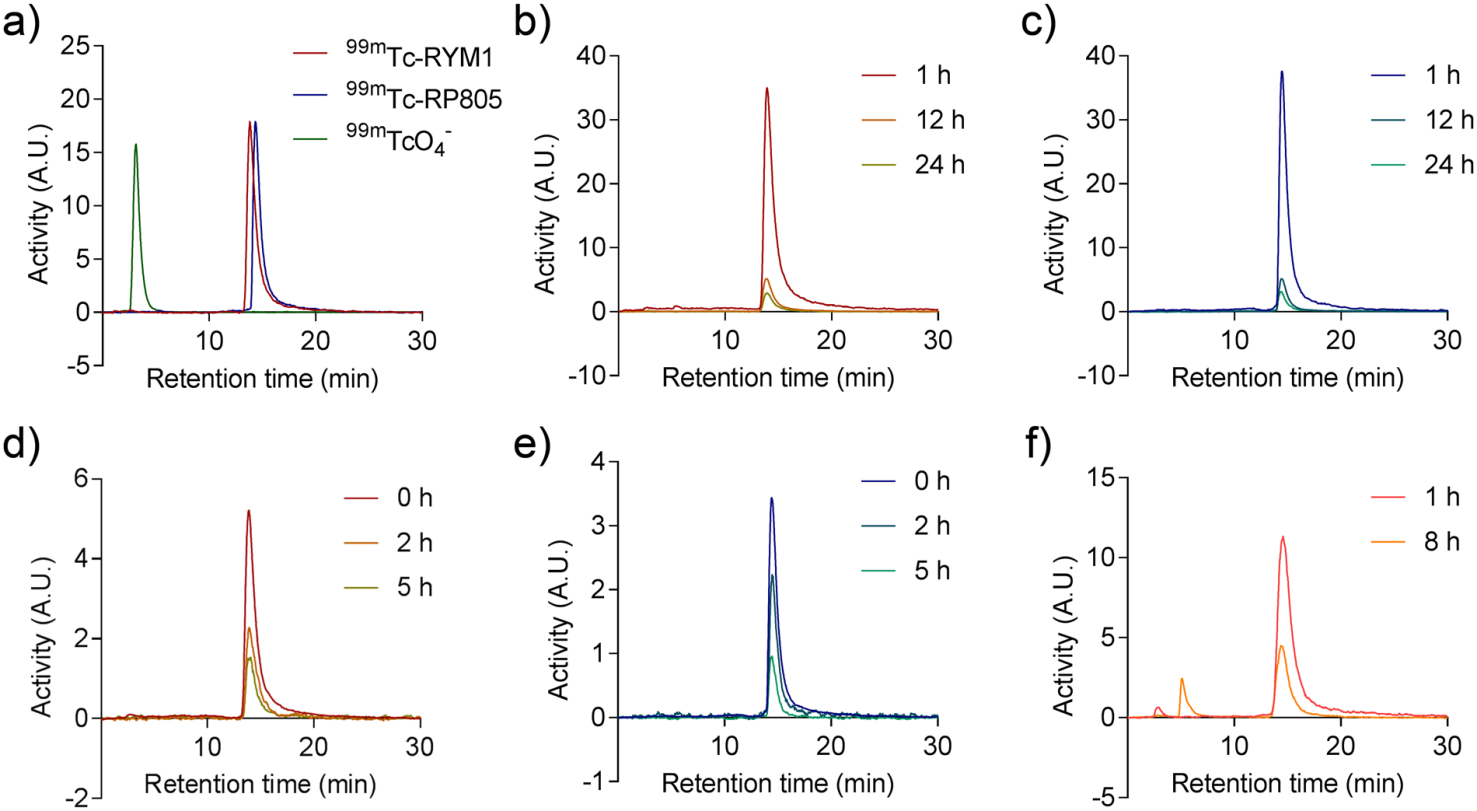
Radio-HPLC analysis of ^99m^Tc-labeled compounds in saline and blood: (**a**) comparison of free ^99m^TcO_4_^−^_,_^99m^Tc-RYM1 and RP805; (**b**) stability of ^99m^Tc-RYM1 in saline; (**c**) stability of RP805 in saline; (**d**) stability of ^99m^Tc-RYM1 in blood; (**e**) stability of RP805 in blood; (**f**) stability of ^99m^Tc-RYM1(tricine)_2_ in saline. A.U.: arbitrary units.

We first labeled RYM1 with ^99m^Tc in a formulation comprised of tricine, mannitol, TPPTS, and pluronic acid in succinate buffer (pH 5.0), as described previously (Fig. 6)^22,29^. For optimizing radiolabeling, we tested a range of RYM1 from 2 to 10 µg (2.1~10.5 nmol, considering 2 associated TFA molecules) in 2–10 µL dimethyl sulfoxide (DMSO) mixed with 200.0 µL vehicle solution, followed by adding 50–100 µL (5.0–10.0 mCi) of ^99m^TcO_4_^−^ solution. The mixture was heated at 95 °C for 10 min and cooled down to room temperature before subjecting it to radio-HPLC analysis. HPLC analysis was performed using aqueous acetonitrile (containing 0.16% ammonium formate) as mobile phase for gradient elution, and showed one single peak of the desired ^99m^Tc-labelled product, i.e. ^99m^Tc-RYM1 at retention time of 13.8 min (Fig. 7a). ^99m^Tc-RYM1 was consistently obtained in a high radiochemical yield and radiochemical purity (>95%) with minimal formation of ^99m^Tc-colloid or ^99m^Tc-co-ligand product (<2%) even adding 10 mCi of ^99m^TcO_4_^−^ solution. Based on the initial activity of 5~10 mCi (185~370 MBq) of ^99m^TcO_4_^−^ and 2 µg (2.1 nmol) RYM1 added in 250~300 μL solution, the specific activity of ^99m^Tc-RYM1 was calculated as 2.4~4.8 mCi/nmol (89~178 MBq/nmol). We also prepared RP805 precursor and used it for ^99m^Tc labeling similarly. Compared with ^99m^Tc-RYM1 at retention time of 13.8 min, RP805 had a longer retention time of 14.6 min.

To further confirm the radiolabeling product, RYM1 was also labeled with tricine and TPPTS in the presence of SnCl_2_ (Fig. 6). The ^99m^Tc-RYM1 samples obtained from both methods had identical retention time in radio-HPLC spectra. In addition, a mixture of ^99m^Tc and the succinate solution without RYM1 was also heated at 90 °C for 10 min. Radio-HPLC analysis showed only one major peak of [^99m^Tc]-colloid or [^99m^Tc]-co-ligand product at 3.3 min but there was no radioactive peak at 13.8 min. Combined, these results confirmed the successful labeling to give ^99m^Tc-RYM1.

One advantage of HYNIC ligand is to allow the use of different co-ligands such as TPPTS, tricine, and EDDA to give various^99m^Tc labeling analogs of different hydrophilicity^30–32^. Therefore, we did ^99m^Tc-labeling of RYM1 in the presence of SnCl_2_ and tricine to give a ^99m^Tc-labeled product which was expected to be ^99m^Tc-RYM1 analog with two tricine co-ligands, i.e. ^99m^Tc-RYM1 (tricine)_2_ (Fig. 6). To this end, a solution of 5 µL/1 mM in DMSO, ^99m^Tc, and tricine-Sn solution was heated at 90 °C for 20 min to give the ^99m^Tc-labeling analog. Radio-HPLC showed the desired radioactive peak at 15.5 min in high radiochemical purity and yield (98%).

### Stability studies

The resulting ^99m^Tc-RYM1 was monitored by the radio-HPLC analysis for stability over 24 h post-labeling. As shown in Fig. 7, both ^99m^Tc-RYM1 and RP805 showed good radiochemical stability in saline buffer at room temperature over 24 h. Both also exhibited good stability in murine blood samples incubated at 37 °C for 2 h (Fig. 7). However, the ^99m^Tc-labeled analog containing two tricine co-ligands, i.e., ^99m^Tc-RYM1(tricine)_2_, showed low stability in saline. The desired peak at 15.5 min was decreased to ~90% after keeping at room temperature for 8 h (Fig. 7f). A new peak at 5.6 min was increased, which might be related with free ^99m^Tc release from ^99m^Tc-RYM1(tricine)_2_, as suggested by radio-HPLC analysis of the ^99m^TcO_4_^−^ solution under the same condition. Based on the ratio of the two radioactive peaks, 90% of ^99m^Tc-RYM1(tricine)_2_ was decomposed when kept at room temperature for 24 h.

### LogP measurements

Compounds RYM and RYM1 have cLogP values of −0.23 and 0.50, respectively. Accordingly, RYM1 has increased hydrophilicity compared with RP805 precursor (cLogP 3.65). The two ^99m^Tc labeled compounds, ^99m^Tc-RYM1 and RP805 were evaluated for their partitions between n-octanol and water or Tris buffer (pH 7.4) (Table 2)^33^. The negative Log values indicated that both ^99m^Tc-labeled compounds were water soluble. Compared with RP805, the lower partition coefficient and shorter retention time in radio-HPLC indicated the improved hydrophilicity of ^99m^Tc-RYM1, as expected.

**Table 2:** LogP values of ^99m^Tc-RYM1 and RP805. The data represent the mean ± SD of 2 independent experiments, each performed with triplicate determinations.

LogP	Octanol/Tris*	Octanol/water
RP805	−3.2 ± 0.1	−2.8 ± 0.0
^99m^Tc-RYM1	−4.4 ± 0.1	−4.0 ± 0.1

*pH 7.4.

### Effects of ^99m^Tc-labeling on MMP binding

**Figure 8:**
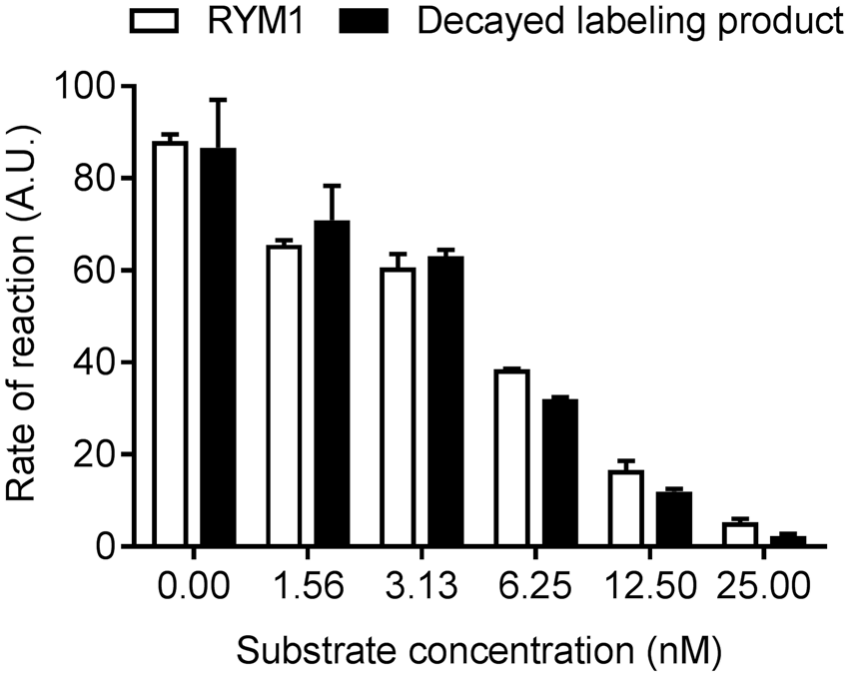
MMP-12 inhibitory activities of RYM1 and decayed RYM1 labeling mixture. The data represent the mean ± SD of duplicate determinations. A.U.: arbitrary unit.

Next, we evaluated the effect of ^99m^Tc-labeling on RYM MMP inhibition, using rhMMP-12 as a representative enzyme. A solution of ^99m^Tc-RYM1 prepared from 2 µg of RYM1 was decayed at −80 °C for two days, lyophilized, and re-dissolved in saline for MMP inhibition assays. The decayed solution (which contains a mixture of the unlabeled precursor (RYM1), the added co-ligands, and the decayed products ^99m^Tc-RYM1) showed similar MMP inhibition potencies with RYM1 based on the fluorescence change kinetics, i.e. relative fluorescence units per min (RFU/min), suggesting that the labeling procedure had no significant effect on MMP binding (Fig. 8).

### Tissue binding *ex vivo*

**Figure 9:**
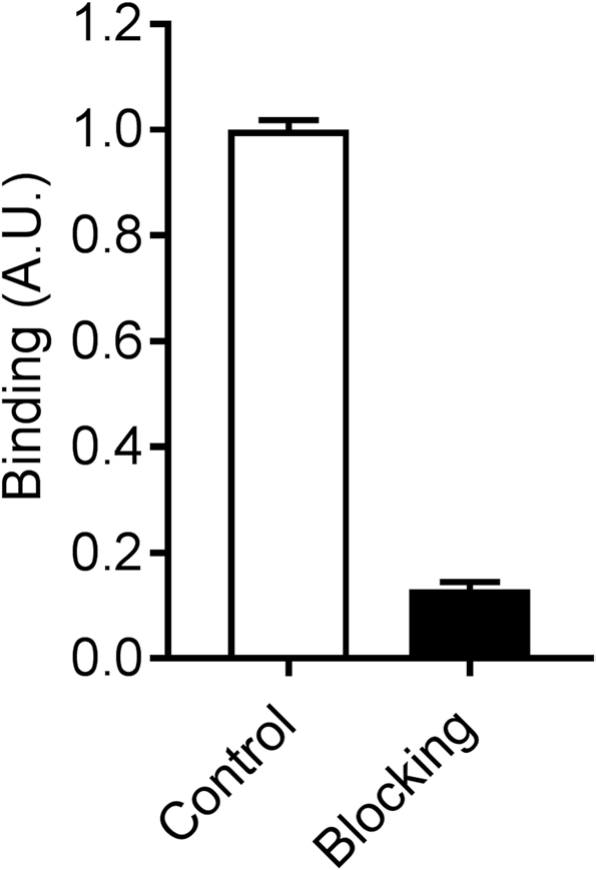
*Ex vivo* binding of ^99m^Tc-RYM1 to lung tissue from lung-specific interleukin-13 transgenic mice without (control) or with (blocking) co-incubation of excess precursor, RYM. The data represent the mean ± SD of duplicate determinations. A.U.: arbitrary unit.

We have shown that the lungs of lung-specific interleukin (IL)-13 transgenic (tg) [Club cell 10-kDa protein (CC10)-IL-13 Tg] mice, a pre-clinical model of pulmonary pathology, express elevated MMP activity in conjunction with tissue remodeling and inflammation^20^. Therefore, as a prelude to *in vivo* imaging studies^23^, we evaluated binding of ^99m^Tc-RYM1 to lung tissue homogenates of CC10-IL-13Tg mice (n = 2). Consistent with the MMP-binding property of ^99m^Tc-RYM1, there was considerable retention of this tracer, which was reduced by 87% upon co-incubation with an excess of RYM, demonstrating its binding specificity in the lung tissue (Fig. 9).

## Discussion

We have designed, synthesized, and evaluated a new zwitterionic anti-succinate-based macrocyclic hydroxamate analog RYM, its HYNIC conjugate RYM1 and related ^99m^Tc labeled analog ^99m^Tc-RYM1. The compounds, which have good water solubility and nanomolar inhibition *in vitro* against a panel of rhMMPs and TACE, represent a new category of dual MMP/TACE inhibitors.

Hydroxamate-based MMP inhibitors such as GM-6001, batimastat (BB-94) and marimastat (BB-2516) bind to the zinc ion of the catalytic domain of activated MMPs for potent broad-spectrum inhibition^2,3,34^. The clinical use of these inhibitors is hampered by toxicity and side effects, which may stem from lack of targeting specificity and limited selectivity. Accordingly, other non-hydroxamate and hydroxamate MMP inhibitors with improved MMP selectivity have been developed, and are undergoing preclinical and clinical evaluation^9,28,35–38^. Despite the limitations of hydroxamate inhibitors as therapeutic agents, these compounds are of value in molecular imaging, which is governed by a different set of criteria. Accordingly, we have used RP805, a ^99m^Tc-labeled hydroxamate tracer, to image MMP activation by SPECT imaging in pre-clinical models of cardiovascular and pulmonary pathology without any evidence of toxicity^17,19–22,39–41^. Prior to this work, RP805 was the only ^99m^Tc-labeled imaging agent that has shown promise in imaging MMP activation in cardiovascular pathology. However, RP805 has a relatively long circulation time, which could be a barrier to vascular imaging. Indeed, given the physical proximity of the blood pool and vessel wall as well as the small size of the arterial wall, imaging in atherosclerosis, aneurysm and other vascular pathologies is critically dependent on the contrast between their signals. A long clearance time would require delayed imaging which can adversely affect the contrast and signal to background ratio. In addition, the limited water-solubility of RP805 precursor is a barrier for establishing the specificity of the MMP signal in blocking studies. This is a key issue, as most cardiovascular pathologies are associated with inflammation and enhanced tissue permeability that promote non-specific uptake of the tracers. Therefore, it is critical to demonstrate signal specificity for further development and translation. We hypothesized that novel hydroxamate-based MMP inhibitors with distinct physicochemical properties, favorable pharmacokinetics and better *in vivo* targeting could be attractive candidates for development of alternative tracers with improved imaging characteristics. Given the paucity of promising MMP-targeting imaging agents and aforementioned limitations of RP805, the development of such rationally-designed, improved MMP tracers would have a major impact on cardiovascular medicine with paradigm-shifting potential.

**Figure 10:**
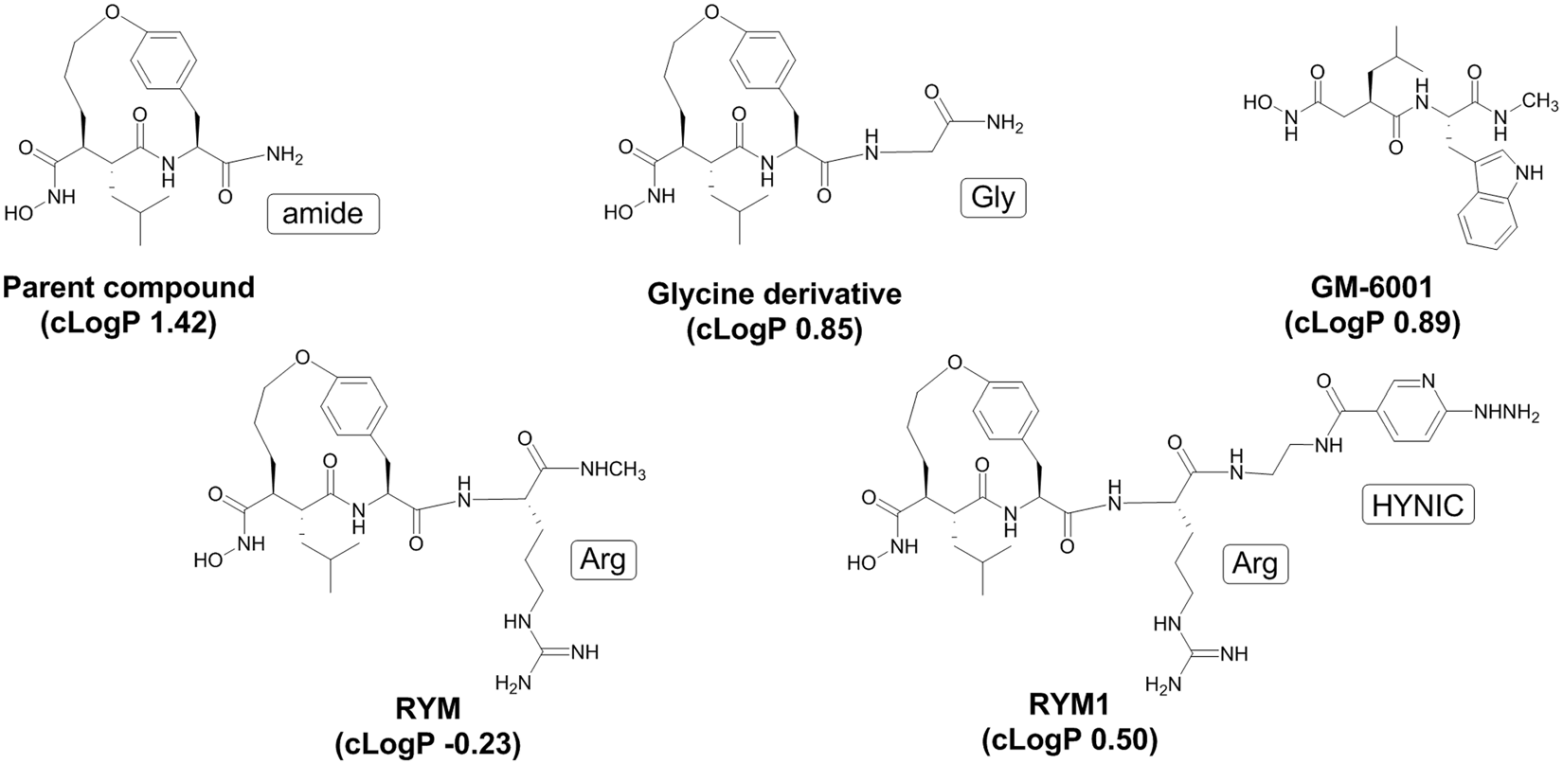
Chemical structures and cLogP values of hydroxamate-based MMP inhibitors.

Water solubility or hydrophilicity is a determinant of a drug governing its formulation, pharmacokinetics and *in vivo* performance. Many MMP inhibitors such as GM-6001 and batimastat (BB-94) have very poor water solubility, and it is reported that glycine incorporation at P3′ is a critical structural component to achieve both good *in vitro* and oral activity. To develop novel water soluble MMP inhibitors, we first calculated the cLogP values GM-6001 and several related compounds using ChemDraw Professional 16.0 (Fig. 10). Compared with GM-6001 (cLogP: 0.89), the RP805 parent macrocyclic hydroxamate amide analog (cLogP: 1.42) and its glycine-containing analog (cLogP: 0.85), incorporation of an Arg side chain was expected to considerably increase the hydrophilicity of the resulting novel analogs. Indeed, both RYM (cLogP: −0.23) and RYM1 (cLogP: 0.50) had relatively low cLogP values. In addition, the positively charged guanidine of Arg and negatively charged hydroxamate groups displayed around the anti-succinate-based macrocycle scaffold show the structural characteristics of a zwitterionic molecule, a feature which may improve *in vivo* distribution and specific targeting^42–44^. Therefore, as a novel approach to improving water-solubility and biodistribution, we synthesized several new Arg-containing macrocyclic hydroxamate analogs, including RYM, its HYNIC conjugate (RYM1) and related ^99m^Tc-labeled analog, ^99m^Tc-RYM1.

Macrocyclic hydroxamate MMP inhibitors can be synthesized starting from anti-succinate derivatives, as reported previously^24,25^. To synthesize new Arg-containing analogs, we used alternative multiple protection and de-protection strategies in 13 and 16 steps (Figs 3, 4 and 5). This novel synthetic strategy included: (1) synthesis of the orthogonally protected macrocyclic intermediate (**I**-**7**), (2) Arg introduction, (3) conversion of carboxylic acid into hydroxamic acid, and (4) HYNIC conjugation. As expected, the Arg-containing macrocyclic hydroxamate MMP inhibitors, RYM and RYM1, showed good water solubility (10 µg/µL). Furthermore, similar to RP805 precursor^16,20^, the compounds displayed nanomolar inhibition *in vitro* against a panel of rhMMPs including rhMMP-2, rhMMP-7, rhMMP-9, rhMMP-12, and rhMMP-13, as well as TACE. The results confirmed our hypothesis that the Arg at P3′ can improve the water solubility greatly but does not perturb the binding of hydroxamate group to the catalytic Zn^2+^ of rhMMPs. K_i_ values of RYM against 5 rhMMPs showed the relative inhibition potency in the following order: rhMMP-12 > rhMMP-2 > rhMMP-7~rhMMP-13 > rhMMP-9. Albeit not high, the rhMMP-12 selectivity of RYM over the other rhMMPs is remarkable compared with GM-6001, one of non-selective potent hydroxamate MMP inhibitors. We deduce that the selectivity may be ascribed to the macrocyclization between α substituent and P2′ substituent which may favor for rhMMP-12 especially^45^. Both RYM and RYM1 had similar K_i_ values against rhMMP-12, indicating the HYNIC motif and related linker might not impact the MMP binding significantly.

Because of the structural similarity between the ADAM and MMP families of proteases, we evaluated the binding of RYM to TACE (ADAM17), as a representative ADAM^25^. TACE is often upregulated and activated (and implicated) in inflammatory disorders, where MMP activation also plays a key role^46^. Accordingly, it is reasonable to speculate that by targeting both TACE and MMP activation, RYM-based tracers may prove to be more effective in tracking inflammation *in vivo*. Direct comparison of these tracers (and selectivity toward other ADAMs) would require dedicated sets of studies that are beyond the scope of this report.

HYNIC has been widely used as a chelator for ^99m^Tc-labeling. It needs two co-ligands such as TPPTS and tricine or two tricine^30–32^. As described for RP805, RYM1 was conveniently labeled with ^99m^Tc in the presence of both TPPTS and tricine in a formulation to form the stable ^99m^Tc-labeling product, ^99m^Tc-RYM1, in high radiochemical purity and yield. The labeling can also be achieved in the presence of SnCl_2_ to give ^99m^Tc-RYM1 by an alternative method. We also confirmed two co-ligands i.e. TPPTS and tricine led to higher radiochemical stability than two tricine co-ligands. Importantly, compounds RYM1 and the decayed solution of ^99m^Tc-RYM1 displayed a similar inhibition profile against rhMMP-12 enzymatic activity, indicating the ^99m^Tc-labeling procedure does not have a significant effect on key macrocyclic hydroxamate motif, its MMP binding and inhibition. Furthermore, ^99m^Tc-RYM1 exhibited specific binding with lung tissue from IL-13 transgenic mice *ex vivo*, which express high levels of MMP activity^20^.

We expected the structural differences between RP805 and ^99m^Tc-RYM1would lead to different biodistribution, pharmacokinetics, and targeting localization. This was empirically demonstrated in murine models, where ^99m^Tc-RYM1 showed faster blood clearance with residual blood activity reduced by more than 50% at 1 h post-injection, and significantly higher specific uptake in several organs relative to RP805^23^. Importantly, in these studies the MMP signal in aneurysm *in vivo* correlated with MMP activity, as determined by zymography *ex vivo*. Similar to aneurysm, MMPs play a key role in the pathogenesis of several pulmonary diseases. We confirmed tissue binding of RYM1 and its specificity in IL-13 Tg lungs, where there is considerable tissue remodeling and inflammation^20^. This is associated with upregulation of several members of MMP family, including MMP-12 and -13, and enhanced MMP-2 and MMP-12 activity. We have shown the feasibility of MMP-targeted imaging with ^99m^Tc-RP85 in this model and demonstrated that the MMP signal correlates with tissue inflammation^20^. A future study comparing ^99m^Tc-RYM1 and ^99m^Tc-RP805 imaging in this model is of interest. However, it is impossible to determine to what extent each specific MMP (or ADAM) contributes to ^99m^Tc-RYM1 (or ^99m^Tc-RP805) binding, as this would require highly selective inhibitors that are currently unavailable. Similarly, MMP gene deletion (even if the animals were available) could alter the lung biology and confound the interpretation of the studies.

Combined, our data suggest that ^99m^Tc-RYM1 is a suitable imaging agent for molecular imaging of MMP activation *in vivo*, especially in vascular pathologies, where rapid blood clearance is critical. While beyond the scope of this manuscript, based on these promising data we are currently working on securing permission for first in-human trial of this tracer. The Arg-containing macrocyclic hydroxamate MMP inhibitors can also serve as lead compounds for structural modification and further development to provide analogs with different linkers and metal chelators for various imaging and therapeutic applications.

### Summary

To address the limitations of existing MMP imaging agents, we have designed, synthesized, and evaluated a new Arg-containing anti-succinate-based macrocyclic hydroxamate analog (RYM), its HYNIC conjugate (RYM1) and related ^99m^Tc labeling analog (^99m^Tc-RYM1). Both RYM and RYM1 displayed nanomolar potent inhibition *in vitro* against a panel of rhMMPs and TACE. RYM1 allows for facile efficient ^99m^Tc-labeling to give its ^99m^Tc-labeled analog, ^99m^Tc-RYM1 in a high radiochemical yield. As a new generation MMP-targeted tracer, ^99m^Tc-RYM1 exhibits specific binding with inflamed lung tissue *ex vivo*. These results, along with favorable *in vivo* imaging studies reported elsewhere^23^, support further development of such water soluble Arg-containing macrocyclic hydroxamate MMP inhibitors with improved physicochemical properties and favorable pharmacokinetics for molecular imaging and potentially, therapeutic applications.

## Materials and Methods

All methods were carried out in accordance with relevant guidelines and regulations.

### Reagents

(2R,3S)-3-(tert-butoxycarbonyl)-2-isobutylhex-5-enoic acid was purchased from Chem-Stone Co., Ltd., Guangzhou, China; Fmoc-Arg(Mtr), L-Tyrosine benzyl ester, and O-(7-Azabenzotriazol-1-yl)-N,N,N′,N′-tetramethyluronium hexafluorophosphate (HATU) were fromChem-Impex International, Inc., Wood Dale, Illinois, USA. Dichloromethane, ethyl acetate, methanol, and acetonitrile were from Avantor Performance Materials, Inc, Center Valley, Pennsylvania, USA. Silica gel (70–90 µm, 60 Å) was purchased from Agela Technologies (Wilmington, Delaware, USA). Other solvents and chemicals were purchased from Sigma-Aldrich, St. Louis, Missouri, USA.

### Instruments

(1) HPLC Waters HPLC system 2489, UV-Vis detector, 600 controller, and Empower Pro software. (2) LC-MS: Agilent LC-MS 6120B Quadrupole, 6550 A iFunnel Q-TOF. (3) NMR: Agilent NMR DD2 400 MHz with OneProbe (Agilent Technologies, Santa Clara, CA, USA). (4) Fluorescent plate reader (BIO-TEK/Synergy HT).

### Synthesis

#### Intermediate 1 (I-1)

To a stirred solution of (2 R,3 S)-3-(tert-butoxycarbonyl)-2-iso-butylhex-5-enoic acid (10.0 g, 95%, 35.1 mmol) and benzyl bromide (15 g, 85.9 mmol) in toluene (40 mL), were added dropwise 13.0 g (86 mmol) 1,8-diazabicyclo[5.4.0]undec-7-ene (DBU) in 20 mL toluene. The resulting mixture was stirred at room temperature for 2 h and then at 60 °C for 1 h. The toluene solution was separated from the precipitated solid residue. The residue was dissolved in water (20 mL), and extracted with ethyl acetate (20 mL × 3). The combined toluene and ethyl acetate solution (100 mL) was washed with 1 N HCl (20 mL × 2), water (20 mL × 2) and brine (20 mL × 2). It was dried over anhydrous MgSO_4_, filtered and concentrated under vacuum. The resulting residue was purified by chromatography (silica gel, 2% ethyl acetate/hexane) to afford 9.0 g (71%) of the title compound as an oil. ES-MS: Observed [MH]^+^ 360.1 and [MNa]^+^ 383.2.

#### Intermediate 2 (I-2)

To a stirred solution of **I-1** (9.0 g, 24.9 mmol) in 30 mL anhydrous THF, at 0 °C, was added dropwise 9-BBN in THF (200 mL, 100 mmol) over a period of 30 min and allowed to stir at room temperature overnight. 10 mL water was added dropwise after the solution was cooled in an ice bath. A solution of 9.9 g NaOAc in water (30 mL) was added, followed by the addition of 30% H_2_O_2_ (30 mL) dropwise. The mixture was stirred at room temperature for 60 min and concentrated under vacuum. The resulting aqueous solution was extracted with ethyl acetate (20 mL × 4). The combined ethyl acetate solution (80 mL) was washed with 1 N HCl (20 mL × 2), water (20 mL × 2), and brine (20 mL × 2). After it was dried over anhydrous MgSO_4_, the solution was filtered and concentrated under vacuum. The resulting residue was purified by silica gel chromatography (silica gel, 20% ethyl acetate/hexane) to give the title compound (7.2 g, 70%). ES-MS: observed [MH-tert-butyl]^+^ 323.0, [MNa]^+^ 401.1, and [MH-tert-butyl-H_2_O]^+^ 305.2.

#### Intermediate 3 (I-3)

To a stirred solution of **I-2** (7.0 g, 19.0 mmol) and CBr_4_ (12.6 g, 38 mmol) in anhydrous DCM (30 mL), was added Ph_3_P (10.4 g, 40 mmol) in small portions for 30 min. The resulting mixture was stirred at room temperature for another 2 h, followed by adding 30 mL hexanes. The resulting mixture was transferred to a short column of silica gel for quick elution using DCM and hexane (1:1). The desired fractions were combined and concentrated to get a crude product which was further purified by column chromatography (silica gel, 2% ethyl acetate/hexane). 5.1 g (63%) of the title compound was obtained. ES-MS: Observed double peak [MNa]^+^ 463.0/465.2 and [M-tert-butyl]^+^ 385.0/387.0.

#### Intermediate 4 (I-4)

A mixture of **I-3** (5.0 g, 11.3 mmol) and Pd/C (2.0 g, 10%, wet) in 30 mL methanol was stirred under hydrogen gas at room temperature for 30 min. The mixture was filtered, followed by washing the Pd/C with methanol (5 mL × 4). The combined methanol filtrate was concentrated and the product (3.3 g, 85%) obtained was used for the next step without further purification. ES-MS: Observed double peak [MNa]^+^ 373.0/375.0, [M-tert-butyl-H_2_O]^+^ 277.0/279.0 (100%), and [MNa-tert-butyl]^+^ 318.0/320.0.

#### Intermediate 5 (I-5)

To a stirred solution of I-4 (3.0 g, 8.5 mmol), HOBT (1.7 g, 12.7 mmol), and Tyr-OBz (3.4 g, 12.7 mmol) in 20 mL anhydrous DMF cooled in an ice bath, was added EDCI (2.4 g, 12.7 mmol) and stirred at room temperature for 2.5 h. The resulting mixture was concentrated under high vacuum, re-dissolved with ethyl acetate (100 mL), and washed with water, 1 N HCl, water, 1 N Na_2_CO_3_ solution, water, and brine. The ethyl acetate solution was dried over anhydrous MgSO_4_, filtered, and concentrated under vacuum. The resulting residue was purified by chromatography (silica gel, 20% ethyl acetate/hexane). 3.8 g (74%) of the title compound was obtained. ES-MS: Observed [MNa]^+^ double peak 626.0/628.0 and [MH]^+^ 604.2/606.0.

^1^H NMR (400 MHz, CDCl_3_) δ 0.8 (6 H, d, *J* = 8 Hz), 1.01 (1 H, m), 1.24 (2 H, m), 1.45 (9 H, s), 1.60–1.77 (4 H, m), 2.36 (2 H, m), 2.97 (1 H, m), 3.09 (1 H, m), 3.21 (1 H, m), 3.35 (1 H, m), 4.97 (1 H, m), 5.16 (2 H, m), 5.50 (1 H, s), 6.01 (1 H, m), 6.50–7.37 (9 H, aromatic H); ^13^C NMR (100 MHz, CDCl_3_) δ 21.52, 23.96, 25.85, 28.21, 29.77, 30.60, 33.28, 37.64, 40.69, 48.42, 49.17, 53.15, 67.54, 81.41, 115.72, 127.70, 128.73, 128.75, 128.79, 130.51, 135.14, 155.09, 171.58, 173.42, 173.68.

#### Intermediate 6 (I-6)

To a stirred solution of Cs_2_CO_3_ (3.2 g, 23.4 mmol) in 500 mL anhydrous acetonitrile at 60 °C, was added dropwise a solution of **I-5** (3.0 g, 5.3 mmol) in 50 mL acetonitrile, over a period of 1 h. The resulting mixture was stirred at 60 °C for another 3 h and concentrated under vacuum. The product was re-dissolved with ethyl acetate and filtered, followed by washing the solid with ethyl acetate for 5 times (10 mL × 5). The combined ethyl acetate filtrate was washed with 1 N HCl solution, water, and brine. The ethyl acetate solution was dried over anhydrous MgSO_4_, filtered, and concentrated. The resulting residue was purified by silica gel chromatography using (silica gel, 20% ethyl acetate/hexane) to give the product (1.6 g, 60%). ES-MS: observed [MH]^+^ 524.2, [MNa]^+^ 546.2, [MH-tert-butyl]^+^ 468, and [MCs]^+^ 656.2; ^1^H NMR (400 MHz, CDCl_3_) δ −0.47 (1 H, m), 0.61 (1 H, m), 0.75 (6 H, m), 0.81 (1 H, m), 1.21–1.55 (4 H, m), 1.37 (9 H, s), 1.86 (1 H, m), 2.02 (1 H, m), 2.52 (1 H, m), 3.57 (1 H, m), 4.04 (1 H, m), 4.2 (1 H, m), 5.11–5.24 (3 H, m), 5.51 (1 H, m), 6.94–7.40 (9 H, aromatic H); ^13^C NMR (100 MHz, CDCl_3_) δ 21.29, 24.05, 25.61, 28.20, 29.93, 31.20, 37.85, 40.55, 49.45, 50.01, 51.61, 67.56, 73.91, 80.81, 120.52, 123.59, 128,67, 128.77, 128.83, 129,87, 131.44, 132.01, 135.21, 159.38, 171.73, 173.11, 174.01.

#### Intermediate 7 (I-7)

A mixture of **I-6** (5.0 g, 9.5 mmol), 10% Pd/C (2.0 g), and HCOONH_4_ (5 g) in 15 mL CH_3_OH was stirred at room temperature for 2–3 h until the hydrogen gas evolved was observed. The mixture was further stirred for another 20 min and filtered, followed by washing the Pd/C with CH_3_OH (5 mL × 4). The methanol filtrate was concentrated and acidified with 1 N HCl solution to get a residue which was extracted with ethyl acetate. The ethyl acetate solution was washed with water (10 mL × 2) and brine (10 mL × 2), dried over anhydrous MgSO_4_, filtered, and concentrated. The product (3.7 g, 90%) was obtained and used for the next step without further purification. LC-MS: Observed [MH]^+^ 434.2 and [MNa]^+^ 456.2.

#### Intermediate 8 (I-8)

A mixture of Fmoc-Arg(Mtr)-OH (1.2 g, 1.97 mmol), HOBT (0.27 g, 2.0 mmol), CH_3_NH_2_ 2 M in THF(2 mL, 4.00 mmol) was dissolved in 5 mL anhydrous DMF and cooled at 0~5 °C in an ice bath, followed by adding EDCI (600 mg, 3.1 mmol). The mixture was stirred at room temperature overnight and concentrated under high vacuum. The residue was triturated with 1 N HCl, the solid obtained was filtered, and further washed with 1 N HCl, 5 N Na_2_CO_3_, and H_2_O. The solid product was dried to give 1.03 g (84%) of the title compound. ES-MS: Observed [MH]^+^ 622.2.

#### Intermediate 9 (I-9)

1.0 g of **I-8** obtained above was dissolved in 10 mL DCM and 3 mL piperidine. The mixture was stirred at room temperature for 30 min and concentrated under vacuum. The product was purified by flash column chromatography (2% Methanol/DCM) to give the title compound (0.54 g, 85%). ES-MS: Observed [MH]^+^ 400.2 and [M-H]^−^ 398.1.

#### Intermediate 10 (I-10)

A mixture of **I-7 (**200.0 mg, 0.46 mmol), **I-9** (219.7 mg, 0.55 mmol), and HOBT (115.0 mg, 0.55 mmol) were dissolved in 5 mL DMF and cooled at 0~5 °C in an ice bath, followed by adding EDCI (100 mg, 0.6 mmol). The mixture was stirred at room temperature overnight and concentrated. The residue was dissolved in 10 mL CH_2_Cl_2_, washed with 1 N HCl, H_2_O, and brine. The DCM solution was dried over MgSO_4_, filtered, and concentrated. The product was purified by flash column chromatography (2% methanol/DCM) to give the title compound (300.0 mg, 80%). ES-MS: Observed [MH]^+^ 815.4.

#### Intermediate 11 (I-11)

250 mg of **I-10** was dissolved in 4 mL TFA and 1 mL DCM. After stirred at room temperature for 2 h, the solution was concentrated and dried under vacuum to get the title compound in a quantitative yield. ES-MS: Observed [MH]^+^ 759.3, [MH_2_]^2+^ 392.1.

#### Intermediate 12 (I-12)

A mixture of **I-11 (**200 mg, 0.26 mmol), HOAT (86.0 mg, 0.6 mmol), HATU (236.0 mg, 0.6 mmol), and DIEA (16.0 mg, 0.9 mmol) was dissolved in 4 mL anhydrous DMF. The mixture was stirred at room temperature for 20 min, followed by adding TBDMS-ONH_2_ (137.0 mg, 0.93 mmol). The mixture was further stirred at room temperature overnight and concentrated. The residue was triturated with 1 N HCl, the solid obtained was filtered, and washed with 1 N Na_2_CO_3_, water and brine. The product was further purified by flash column chromatography to give 100.0 mg (50%) of the title compound.

#### Target compound, RYM

**I-12** (50 mg, 0.065 mmol) was dissolved in a mixture of 9.0 mL TFA, 0.5 mL H_2_O, 0.25 mL TIS, and 0.25 g phenol. The mixture was stirred at room temperature overnight and concentrated under vacuum. The residue was added into 10 mL cooled diethyl ether. The precipitated product was collected by centrifugation and purified by semi-preparative HPLC using an eluent of aqueous acetonitrile. The desired fractions were collected and lyophilized to give the title compound (10.0 mg, 22%) as confirmed by both LC-MS and analytical HPLC. ES-MS: Observed [MH]^+^ 562.3, [MH_2_]^2+^ 281.7, and [M-H]^−^ 560.2; ^1^H NMR (400 MHz, CDCl_3_) δ 0.8 (3 H, dd, *J* = 6.6, 2.6 Hz), 0.85 (3 H, d, *J* = 6.5 Hz), 1.46–1.22 (4 H, m), 1.79–1.55 (2 H, m), 1.85 (1 H, dq, *J* = 9.8, 6.7 Hz), 2.17 (1 H, td, *J* = 11.1, 3.4 Hz), 2.77–2.60 (1 H, m), 3.21 (2 H, p, *J* = 6.7 Hz), 3.39 (1 H, dt, *J* = 13.6, 4.4 Hz), 4.24–4.07 (2 H, m), 4.38 (1 H, ddd, *J* = 13.8, 8.0, 5.7 Hz), 4.38 (1 H, ddd, *J* = 13.8, 8.0, 5.7 Hz), 5.03–4.89 (1 H, m), 6.91 (1 H, dt, *J* = 8.3, 2.7 Hz), 7.09 (1 H, dd, *J* = 8.3, 2.4 Hz), 7.17 (1 H, dd, *J* = 8.3, 2.2 Hz), 7.26 (1 H, dd, *J* = 8.4, 2.2 Hz), 8.30 (1 H, dd, *J* = 23.6, 9.7 Hz); ^13^C NMR (100 MHz, CDCl_3_) δ 20.27, 23.08, 24.97, 25.60, 29.33, 29.53, 29.92, 36.52, 40.10, 40.10, 40.60, 46.95, 47.17, 47.38, 47.59, 47.66, 47.81, 47.88, 48.02, 48.23, 52.73, 53.58, 73.15, 120.17, 122.42, 128.88, 132.38, 157.26, 158.99, 171.34, 172.10, 172.56, 174.81.

#### HYNIC

A mixture of 6-chloronicotinic acid (10.0 g, 63.4 mmol), hydrazine (20 mL), and water (20 mL) was refluxed at 95~100 °C for 4 h. The mixture was concentrated under vacuum and the residue was dissolved in 40 mL water. The resulting solution was acidified with 1 N HCl to reach a pH 5~5.5. The solution was kept in a refrigerator overnight. The precipitated solid was collected by filtration, washed with cold water (10 mL × 2) and ether (10 mL × 2). The solid was dried to give 8.5 g (87%) of the title compound. ES-MS: Observed [MH]^+^ 154.1.

#### Boc-HYNIC

A mixture of 4.0 g HYNIC and 8.0 g Na_2_CO_3_ in 60 mL of 1,4-dioxane and 60 mL of water was cooled in an ice bath. 8.2 g of Boc_2_O was added, and stirred at room temperature for 5 h. The mixture was concentrated and the residue was acidified with 1 N HCl solution, the precipitated solid was collected by filtration and dried to give the title compound.

#### Intermediate 13 (I-13)

A mixture of Fmoc-Arg(Mtr)-OH (1.2 g, 1.97 mmol), HOBT (0.32 g, 2.36 mmol), and NH_2_CH_2_CH_2_NHBoc (0.38 g, 2.36 mmol) were dissolved in 5 mL anhydrous DMF and cooled at 0~5 °C in an ice bath, followed by adding 543.0 mg EDCI (2.83 mmol). After stirred at room temperature overnight, the mixture was concentrated under high vacuum. The residue was triturated with 1 N HCl, filtered, and washed with 1 N HCl, 1 N Na_2_CO_3_, and H_2_O. The solid product was dried to give 1.3 g (90%) of the title compound. ES-MS: Observed [MH]^+^ 529.6.

#### Intermediate 14 (I-14)

**I-13** (1.0 g, 1.33 mmol) was dissolved in 7 mL DCM and 3 ml piperidine. After stirred at room temperature for 30 min, the mixture was concentrated under high vacuum. The residue was purified by flash column chromatography using DCM and methanol to give 0.52 g (75%) of the title compound. ES-MS: Observed [MH-Boc]^+^ 429.2.

#### Intermediate 15 (I-15)

A mixture of **I-14 (**200.0 mg, 0.46 mmol), H-Arg(Mtr)-NHCH_2_CH_2_NH-Boc (291.0 mg, 0.55 mmol), and HOBT (74.8 mg, 0.55 mmol) was dissolved in 5 mL DMF and cooled at 0~5 °C in an ice bath, followed by adding EDCI (126.5 mg, 0.66 mmol). The mixture was stirred at room temperature overnight and concentrated. The residue was dissolved in 10 mL CH_2_Cl_2_, washed with 1 N HCl, H_2_O, and brine. The DCM solution was dried over MgSO_4_, filtered, and concentrated. The product was purified by flash column chromatography using DCM and methanol to give 260 mg (60%) of the title compound. ES-MS: Observed [MH-Boc]^+^ 845.8 and [MH_2_-tert-butyl]^2+^ 394.7.

#### Intermediate 11 (I-16)

**I-15** (0.2 g, 0.21 mmol) was dissolved in 8 mL TFA and 2 mL DCM. After stirred at room temperature for 2 h, the solution was concentrated and dried under vacuum to give 0.18 g (98%) of the title compound. ES-MS: Observed [MH]^+^ 788.3 and [MH_2_]^2+^ 394.8.

#### Intermediate 12 (I-17)

**I-16** (0.15 g, 0.16 mmol) was dissolved in 5 mL dioxane and 5 mL 1 N Na_2_CO_3_ solution. The mixture was stirred in an ice bath, followed by adding (Boc)_2_O (70.0 mg, 0.32 mmol). After stirring at room temperature for 2 h, the solution was concentrated and the residue was acidified with 1 N HCl to pH 3. The product was extracted with DCM, washed with water, and dried over MgSO_4_. The filtrate was concentrated to give the title compound (135.0 mg, 95%). ES-MS: Observed [MH]^+^ 888.3

#### Intermediate 13 (I-18)

A mixture of **I-17 (**100.0 mg, 0.11 mmol), HOAT (30.4 mg, 0.22 mmol), HATU (83.6 mg, 0.22 mmol), and DIEA (57.0 mg, 0.44 mmol) were dissolved in 5 mL anhydrous DMF. The mixture was stirred at room temperature for 20 min, followed by adding TBDMS-ONH_2_ (147.2 mg, 1.0 mmol). The mixture was stirred at room temperature overnight and concentrated under vacuum. The residue was triturated with 1 N HCl, filtered, and washed with water, 1 N Na_2_CO_3_, and brine. The product was further purified by column chromatography to give 30.0 mg (30%) of the title compound. ES-MS: Observed [MH]^+^ 903.2 and [MNa]^+^ 925.4.

#### Intermediate 14 (I-19)

**I-18** (20 mg, 0.022 mmol) was dissolved in a mixture of 9.0 mL TFA, 0.5 mL H_2_O, 0.25 mL TIS, and 0.25 g phenol. The mixture was stirred at room temperature overnight and concentrated under vacuum. The residue was added into 10 mL cooled diethyl ether. The precipitated product was collected by centrifugation and purified by semi-preparative HPLC using an eluent of aqueous acetonitrile. The desired fractions were collected and lyophilized to give the title compound (5.4 mg, 30%). ES-MS: Observed [MH]^+^ 591.4, [MH_2_]^2+^ 296.3, and [M_2_H_3_]^3+^ 394.6.

#### Intermediate 20 (I-20)

A mixture of **Boc-HYNIC** (10.0 mg, 0.039 mmol), HOAT (5.5 mg, 0.039 mmol), and HATU (15.0 mg, 0.039 mmol) was dissolved in 1 mL anhydrous DMF, followed by adding DIEA (10.0 mg, 0.078 mmol). The mixture was stirred at room temperature for 30 min. To the resulting solution was added a solution of **I-19 (**16.0 mg, 0.0195 mmol) and DIEA (5 µL, 3.71 mg, 0.028 mmol) in 0.5 mL anhydrous DMF. The resulting solution was stirred at room temperature for 1 h and concentrated under vacuum. The residue was triturated with 1 N HCl, filtered, and washed with 1 N Na_2_CO_3_ and water. The solid product collected was used in the next reaction without further purification. ES-MS: Observed [MH]^+^ 826.2 & [MH_2_]^2+^ 413.6.

### RYM1, ((6 S,7 R,10 S)-N^10^-((S)-5-guanidino-1-((2-(6-hydrazinylnicotinamido)ethyl) amino)-1-oxopentan-2-yl)-N^6^-hydroxy-7-isobutyl-8-oxo-2-oxa-9-aza-1(1,4)-benzenacyclo undecaphane-6,10-dicarboxamide)

**I-20** was dissolved in a mixture of 4.5 mL TFA, 0.25 mL H_2_O, 0.125 mL TIS, and 0.125 g phenol. The mixture was stirred at room temperature for 30 min and concentrated under vacuum. The residue was added into 10 mL cooled diethyl ether. The precipitated product was collected by centrifugation and purified by semi-preparative HPLC using an eluent of aqueous acetonitrile. The desired fractions were collected and lyophilized to give the title compound (3.0 mg, 16%) as confirmed by both LC-MS and analytical HPLC. ES-MS: Observed [MH]^+^ 726.3 and [MH_2_]^2+^ 363.7.

### MMP and TACE inhibition assays

The recombinant human MMPs (rhMMPs) including rhMMP-2, rhMMP-7, rhMMP-9, rhMMP-12, and rhMMP-13 were purchased from R&D Systems (Minneapolis, MN, USA). A buffer consisting of 50 mM Tris, 10 mM CaCl_2_, 150 mM NaCl, 0.05% Brij-35 (w/v) (pH 7.5, 1,3,5-Trichloro-2-nitrobenzene) was used for all the MMP assays. A mixture of the rhMMPs (500 ng, 9 µL in assay buffer) and p-aminophenylmercuric acetate (APMA, 1 µL, 10.0 mM in DMSO) was incubated at 37 °C (1 h/rhMMP-2, 2 h/rhMMP-7, 24 h/rhMMP-9, 4 h/rhMMP-12, and 2 h/rhMMP-13) according to the procedures described by R&D Systems. The activated MMPs were diluted to 500 µL in the assay buffer for inhibition assays. The assays were carried out in 96-well black non-binding surface microplates (Fisher Scientific). Seventy microliter (for inhibitor wells) and 80 µL (control wells) of the assay buffer were first added into each well as designed, followed by adding 10 µL (10 ng) of the activated MMP solutions into each well. The inhibitor solutions at 4 different concentrations (10 µL each well) were added into the corresponding inhibitor wells in the microplate which was then swirled at room temperature for 30 min. Ten microliter of the fluorogenic MMP substrate solutions (R&D SYSTEMS®) at 4 different concentrations (20.0, 30.0, 40.0, and 50.0 µM) in assay buffer were added into each well to make a total volume of 100 µL. The microplate fluorescence was measured at 320 nm (excitation) and 405 nm (emission) wavelengths for 30 min (1.44 min intervals, sensitivity 100, shaking intensity 4 and duration 30 s) by a fluorescent plate reader (BIO-TEK/Synergy HT) to generate the fluorescence kinetic curves and related mean velocity values (*v*), i.e. relative fluorescence units per min (RFU/min). Finally, the competitive inhibition constants (*K*_i_ value) were calculated from the mean velocity values, and related substrate (*S*) and inhibitor (*I*) concentrations using GraphPad Prism 6 by the following equation for competitive inhibition, where ${V}_{{\max }}$ is the maximal velocity, the ${K}_{M}$ Michaelis-Menten constant:

$$v=\frac{{V}_{{\max }}[S]}{{K}_{M}(1+\frac{[I]}{{K}_{i}})+[S]}$$

TACE inhibition was evaluated similarly. Recombinant Human TACE/ADAM17 and a fluorogenic peptide substrate i.e. Mca-P-L-A-Q-A-V-Dpa-R-S-S-S-R-NH_2_ (R&D SYSTEMS^®^) were purchased from R&D Systems (Minneapolis, MN). A buffer consisting of 25 mM Tris, 2.5 μM ZnCl_2_, 0.005% Brij35 (w/v) (pH 9.0) was used for TACE inhibition assays.

#### Radiolabeling with ^99m^Tc and HPLC analysis

RYM1 was labeled with ^99m^Tc by heating it with ^99m^TcO_4_ in a vehicle solution containing tricine (6.5 mg/ml), 3,3′,3″-phosphanetriyltris(benzenesulfonic acid) trisodium salt (TPPTS) (5.0 mg/ml), pluronic F-127 (0.1 mg/ml), succinic acid (12.7 mg/ml), sodium succinate (38.5 mg/ml) and Mannitol (40 mg/ml) in high purity and yield. Typically, 2 µg of RYM1 in 2.0 µL (1 mM) DMSO was mixed ~10 mCi/100 µL ^99m^TcO_4_, followed by adding 200 µL of the vehicle solution in a vial. The mixture was heated at 95 °C for 10 min, and cooled to room temperature to yield the ^99m^Tc-labeled product, ^99m^Tc-RYM1, as analyzed by radio-HPLC. Radio-HPLC analysis was performed using Waters RP-HPLC (Milford, MA) on a C-18 reverse-phase analytical column (Phenomenex, Jupiter® 4 µm Proteo 90 Å, 250 × 4.6 mm, Torrance, CA). The mobile phase consisted of Solvent A (aqueous solution containing 25 mM ammonium formate) and solvent B (90% aqueous CH_3_CN containing 25 mM ammonium formate). The gradient was initiated at a flow rate of 1 mL/min and from 90.0 A% for 2 min, followed by a linear gradient to 30% A over 15 min and isocratic run for another 5 min at a flow rate of 1 mL/min over 40 min. UV chromatograms were monitored at both 254 nm and 214 nm. For stability studies, the ^99m^Tc-labeled product was similarly monitored for degradation by RP-HPLC. As analyzed by HPLC under such a condition, ^99m^Tc-RYM1 showed a single peak at retention time of 13.8 min.

#### LogP measurement

An aliquot (10.0 µL/~100 µCi) of RP-805 or ^99m^Tc-RYM1 was added into a mixture of 500.0 µL octanol and 500.0 µL water or tris solution (1 M, pH 7.4) in a 1.5 mL Eppendorf vial. The vial was mixed by vortex violently for 3 min and then centrifuged at 13,000 rpm for 5 min. The two phases i.e. octanol and aqueous layers separated were obtained carefully using a micropipette. The two solutions were centrifuged at 13,000 rpm for 5 min. The samples (10.0 µL) taken from the octanol layer and the aqueous layer were measured for radioactivity in triplicates by r-counter (PerkinElmer, Waltham, MA). The logP_octanol/water_ and logP_octanol/tris_ values were calculated by the following equation: logP = log (decay-corrected radioactivity in octanol sample/decay-corrected radioactivity in aqueous sample).

#### Tissue binding

*Ex vivo* binding of ^99m^Tc-RYM1 to biological tissue was evaluated in lung tissue homogenate of CC10-IL-13Tg mice (n = 2) showing elevated basal MMP activity^20^. Lung tissue (247 ± 85 µg, in duplicates was incubated with 3 nM of ^99m^Tc-RYM1 for 60 min at 37 °C with or without the preincubation of 20 µM of RYM. After tracer incubation, tissue homogenate was spin down and washed 3 times with PBS, before resuspension in protein lysis buffer, gamma-counting (Wizard2, PerkinElmer) and protein concentration was measured (Pierce BCA Protein Assay Kit, Thermo Fisher Scientific and Multiskan Ascent Microplate Photometer, Thermo Labsystems) in order to determine the amount of tissue used. All experimental protocols were approved by Yale University and VA Connecticut Institutional Animal Care and Use Committees.

### Data availability

The datasets generated during and/or analyzed during the current study are available from the corresponding author on reasonable request.

## Electronic supplementary material

Supplemental Figure
