# Supplementary material for: Novel Arginine-containing Macrocyclic MMP Inhibitors: Synthesis, 99mTc-labeling, and Evaluation
Source: Sci Rep. 2018 Aug 3;8:11647. doi: 10.1038/s41598-018-29941-2 (PMC6076275; doi:10.1038/s41598-018-29941-2)

Novel Arginine-containing Macrocyclic MMP Inhibitors: Synthesis, ^{99m}Tc-labeling, and Evaluation

Yunpeng Ye, PhD[1, 2*], Jakub Toczek, PhD[1, 2*], Kiran Gona, PhD[1, 2], Hye-Yeong Kim, PhD[1, 2], Jinah Han, PhD[1, 2], Mahmoud Razavian, PhD[1, 2], Reza Golestani, MD, PhD[1, 2], Jiasheng Zhang, MD, [1, 2], Terence L. Wu, PhD[3], Mousumi Ghosh, PhD[3], Jae-Joon Jung, PhD[1, 2], Mehran M. Sadeghi, MD[1, 2]

1. Cardiovascular Molecular Imaging Laboratory, Section of Cardiovascular Medicine and Yale Cardiovascular Research Center, Yale University School of Medicine, New Haven, CT (USA); 2. Veterans Affairs Connecticut Healthcare System, West Haven, CT (USA); 3. Yale West Campus Analytical Core, Yale University, West Haven, CT (USA)

*: Equally contributed to this project

Supplemental Figure: High resolution mass spectroscopy (HR-MS) of RYM (top) and RYM1 (bottom).

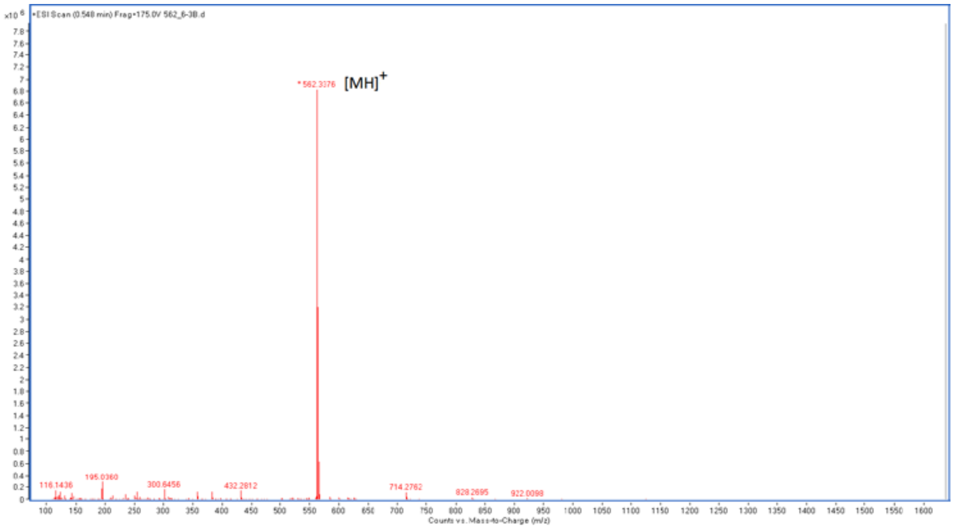

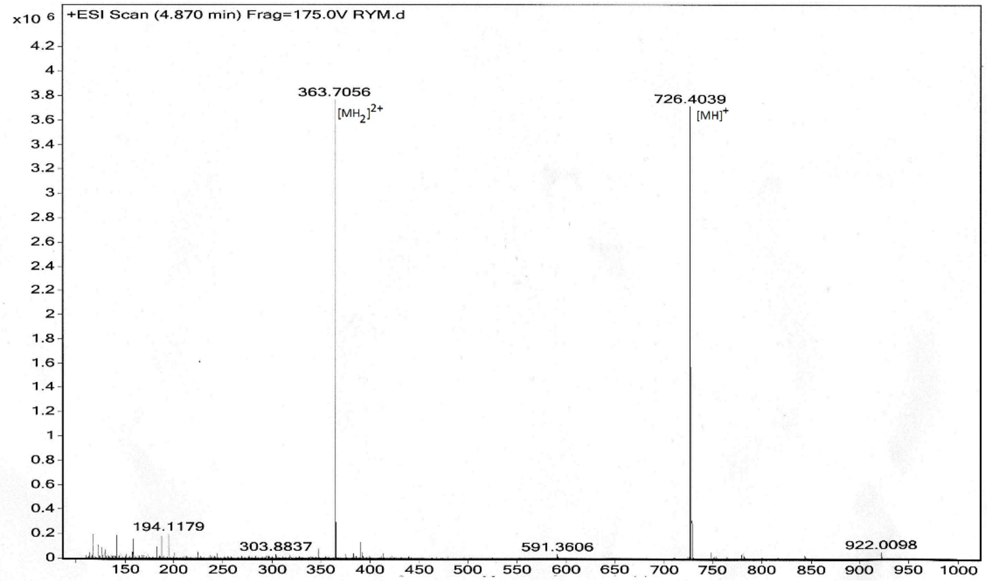

Supplement: Supplementary file 1 — Supplemental Figure [file 41598_2018_29941_MOESM1_ESM.pdf]
